# Time-calibrated phylogeny of neotropical freshwater fishes

**DOI:** 10.3389/fbinf.2024.1433995

**Published:** 2024-12-06

**Authors:** Victor A. Tagliacollo, Milton Tan, Roberto E. Reis, Ronielson Gaia, Virgilio Carrijo, Marcelo Ranuzi, Jack M. Craig, James S. Albert

**Affiliations:** ^1^ Institute of Biology, Federal University of Uberlândia, Uberlândia, Minas Gerais, Brazil; ^2^ Illinois Natural History Survey, University of Illinois at Urbana-Champaign, Champaign, IL, United States; ^3^ Pontifical Catholic University of Rio Grande do Sul, Porto Alegre, Rio Grande do Sul, Brazil; ^4^ Institute for Genomics and Evolutionary Medicine, Temple University, Philadelphia, PA, United States; ^5^ Department of Biology, University of Louisiana at Lafayette, Lafayette, LA, United States

**Keywords:** neotropics, freshwater fishes, phylogeny, divergence times, macroevolution

## Abstract

Neotropical Freshwater Fish (NFF) fauna exhibits the greatest phenotypic disparity and species richness among all continental aquatic vertebrate faunas, with more than 6,345 species distributed across the mostly tropical regions of Central and South America. The last two decades have seen a proliferation of molecular phylogenies, often at the species level, covering almost all 875 valid NFF genera. This study presents the most comprehensive genome-wide, time-calibrated phylogenetic hypothesis of NFF species to date, based on DNA sequences generated over decades through the collaborative efforts of the multinational ichthyological research community. Our purpose is to build and curate an extensive molecular dataset allowing researchers to evaluate macroevolutionary hypotheses in the NFF while facilitating continuous refinement and expansion. Using thousands of DNA sequences from dozens of studies, we compiled a supermatrix of 51 markers for 5,984 taxa, representing 3,167 NFF species. Based on this dataset, we built the most species-rich time-calibrated phylogeny of the NFF taxa to date, summarizing the collective efforts of the ichthyological research community since the midpoint of the last century. We provide a summary review of this remarkable evolutionary history and hope this dataset provides a framework for forthcoming studies of the NFF fauna, documenting compelling, emergent patterns in the world’s most diverse continental vertebrate fauna.

## Introduction

The Neotropics is a vast biological realm encompassing over 17 million km^2^ from Central Mexico to southern Argentina, home to some of the world’s largest rivers, including the Amazon, Orinoco, and La Plata (Lévêque et al., 2008; Van der Sleen and Albert, 2022; Dagosta et al., 2024). These rivers harbor the most diverse continental aquatic vertebrate fauna on Earth, an astonishing diversity of freshwater fishes totaling over 6,345 valid species at present, with more discovered every year (Reis et al., 2016; Albert et al., 2020; Chamon et al., 2022; Becker et al., 2023; Dagosta et al., 2024). Among these are many families found nowhere else on earth, such as the piranha (Serrasalmidae) and electric eel (Gymnotidae), lending the Neotropical Freshwater Fish (NFF) fauna a readily identifiable character and a unique place among the freshwater faunas of the world (Albert et al., 2011).

A large majority (77%) of NFF species diversity is concentrated in just five taxonomic orders (Albert et al., 2011); Characiformes, Cichliformes, Cyprinodontiformes, Gymnotiformes, and Siluriformes. The NFF fauna evolved in isolation for tens of millions of years when South America was an island continent, between its final separation from Africa c. 100 Ma and Antarctica (c. 34 Ma) to its connection with Central America c. 12–3.5 Ma. Over these millions of years, the evolutionary history of NFF taxa was shaped by dynamic processes of river capture (Tagliacollo et al., 2015; Albert et al., 2018; Boschman et al., 2023) and marine incursions (Lovejoy et al., 1998; Bloom et al., 2011; Abreu et al., 2020) at continental scales. This process gave rise to the greatest variety of phenotypes and ecological traits of any continental fish fauna on Earth (Su et al., 2019; Albert et al., 2020). The macroevolutionary patterns in the NFF fauna therefore serve as a compelling example of how continental faunas diversify over evolutionary time periods (Miller and Román-Palacios, 2021), driven by the interactions between intrinsic organismal traits and environmental factors like plate tectonics and climate variability (Silva et al., 2016; Cassemiro et al., 2023; Val et al., 2022).

Yet studying this evolutionary history is challenging as, with more than 6,345 species, inferring comprehensive and accurate relationships among NFF clades is expensive in time and computational resources regardless of the datasets or methods used for phylogeny estimation. This challenge is compounded by the phenotypic diversity of these fishes, which exhibit a myriad of morphological, physiological and behavioral adaptations (Crampton, 2011; Val and Almeida-Val, 2012; Albert et al., 2020; Soares et al., 2023). Nevertheless, substantial molecular data have been produced by the ichthyological research community across hundreds of studies and are now available in public repositories (e.g., Musilová et al., 2008; Cramer, et al., 2011; Oliveira et al., 2011; Thomaz et al., 2015; Tagliacollo et al., 2016; Bragança and Costa, 2018). These data give us an opportunity to build a large-scale, time-calibrated phylogeny as a framework for future evolutionary studies documenting macro-scale patterns in the world’s most diverse continental vertebrate fauna.

Such a phylogeny will help us disentangle the complexity that makes the Neotropics the most species-rich region for freshwater fish on Earth in several ways (Cerezer et al., 2023; Cassemiro et al., 2023). A comprehensive phylogenetic tree is essential for studying evolutionary patterns, offering insights into speciation (Albert et al., 2020), adaptive radiation (López-Fernández, 2021), and lineage diversification (Miller and Román-Palacios, 2021). Considering a phylogenetic tree in the context of major earth history events further reveals the historical distributions of lineages coexisting under similar geological or trophic conditions (Emerson and Gillespie, 2008; Baumsteiger et al., 2012). A phylogeny will also enable large-scale macroevolutionary analyses by facilitating the investigation of rate changes over time (Graham et al., 2018), morphological variations across distantly related lineages (Winemiller, 1991; Jablonski, 2017), and other macroevolutionary patterns (Henao Diaz et al., 2019). Comprehensive phylogenetic trees can even inform conservation strategies by clarifying diversity patterns and identifying unique lineages within regions of high evolutionary diversity (Albert et al., 2020). These insights are invaluable for prioritizing conservation efforts (Dagosta et al., 2021).

To this end, we build on the work of dozens of ichthyologists spanning over three decades of taxonomic, phylogenetic and biogeographical research to present a comprehensive, time-calibrated phylogeny of NFF diversity. The purpose of this study is not primarily to introduce new evolutionary hypotheses or propose new taxonomic classifications, but to compile and systematically organize an extensive, reproducible, and extensible dataset of DNA sequences, alignments, matrices, and phylogenetic trees into a single resource. This is a collective effort, integrating genetic alignments, phylogenetic trees, and divergence time estimates to summarize our current understanding of the evolutionary history of the most diverse continental ichthyofauna on Earth. We aim to continue refining and expanding the dataset, enabling future studies to test alternative evolutionary hypotheses in the NFF. By elucidating NFF relationships on a broad scale, this study establishes a framework for further research and opens new avenues in historical biogeography, macroecology, and macroevolution within the Neotropical ichthyofauna.

## Methodology

### NFF species

The discovery of new species and the reclassification of existing ones are essential processes for refining our understanding of biodiversity and species boundaries. This taxonomic evolution inevitably makes constructing an up-to-date phylogeny challenging. We compiled the list of all valid NFF species described up to December 2021 using information available from original descriptions (Thimotheo et al., 2020; Malabarba et al., 2021), taxonomic reviews (e.g., Crampton et al., 2016; Villa-Navarro et al., 2017; Ferrer and Malabarba, 2020), checklists (Ferraris et al., 2017; Beltrão et al., 2019; Meza-Vargas et al., 2021), and online catalogs (e.g., Eschmeyer’s Catalog of Fishes) (Fricke, 2021). In total, we compiled a list of 6,345 species, 870 genera and 97 families.

### Acquiring DNA sequences

We used the R package PhylotaR (Bennett et al., 2018) to extract published DNA sequences from metadata repositories such as GenBank. PhylotaR is a pipeline designed to automate the retrieval of orthologous DNA sequences, enabling the construction of large-scale phylogenetic trees. It clusters sequences into operational taxonomic units (OTUs) based on predefined taxonomic ranks, such as families, genera, or species, and organizes them by gene region. We used PhylotaR to retrieve sequences for each OTU across all loci from GenBank, downloading them at the family level, using the GenBank Taxonomy Database as a reference (Schoch et al., 2020). To mitigate potential errors from taxonomic misidentification or low-quality sequencing, we retrieved a maximum of three sequences (individuals) per species, when available, and visually inspected the alignments. The vast majority (over 95%) of sequences ascribed to the same species exhibited near-zero nucleotide variation, strongly indicating that the individual sequences were correctly identified, and we therefore retained these in the alignment. In cases where multiple sequences purporting to represent the same species showed high nucleotide variation, suggesting issues related to taxonomic misidentification or sequencing errors, an outlier could be easily identified on the basis of poor homology or imprecise identification (e.g., names including “*cf.*”, “*sp*.” and “*aff.*”), and these were excluded. This approach allowed us to detect and exclude outliers, mitigating aberrant phylogenetic signals due to poor data curation. A similar approach has been applied successfully in other phylogenetic studies (e.g., Arce-H et al., 2017; Mirande, 2019).

PhylotaR retrieved DNA sequences from GenBank through BLAST searches, identifying 51 orthologous clusters, resulting in 43,850 bp, 21,223 sequences, 5,984 terminals, and 3,167 species representing 741 genera (or 86% of NFF genera). The retrieved DNA markers consisted of 61% mitochondrial and 39% nuclear genomic sequences, primarily comprising protein-coding genes and a few non-coding genomic regions (e.g., tRNAs; rRNAs) many of which have been commonly sequenced for phylogenetic research (e.g., Li et al., 2007; Oliveira et al., 2011; Tagliacollo et al., 2012; Ochoa et al., 2017; Melo et al., 2018; Fontenelle et al., 2021). Since orthologous sequences were independently retrieved at the family level, some markers were available for certain families but not for others. Nonetheless, all families showed some degree of marker overlap, ensuring reliable empirical estimates of species evolutionary relationships. To elucidate relationships at finer taxonomic scales, we split the D-LOOP marker into alignments for three specific clades: Siluriformes, Poecilidae, and Cichlidae. This strategy guaranteed a more reliable hypothesis of homologies for this rapidly evolving genetic marker. The complete list of accessions can be found in the Supplementary Material, located in the “csv” folder, under the filename “Annex_1 - accession_numbers.xlsx”.

### Alignment, trimming and partitioning scheme

After downloading the DNA sequences from GenBank, we individually aligned each genetic marker using MAFFT v. 7 (Katoh and Toh, 2008) with default parameters. To enhance data quality, we conducted trimming with GBlocks (Castresana, 2000; Talavera and Castresana, 2007) to remove gap-rich sites at both ends of the alignments, thereby avoiding subjective manual trimming. We achieved this by setting the “Allowed Gap Positions” parameter to -b5 = h, which permits gaps in internal positions but removes them from the flanking regions. This approach ensures that Gblocks eliminates gaps in non-conserved areas while retaining gaps in more conserved regions of the alignment. We concatenated the trimmed alignments into a supermatrix, assigning each marker to an independent partition and GTR evolutionary model with gamma (G) in four rate categories to account for rate heterogeneity among sites. With PartitionFinder2 (Lanfear et al., 2017), we concurrently estimated an optimal partitioning scheme and identified the best-fitting models of molecular evolution for selected partitions. The best-fitting scheme for the supermatrix estimated 30 independent partitions, with each adhering to a GTR + G model. While the partitions share the GTR + G models, each has an independent set of parameter estimates. Alignments both before and after GBocks analysis and partition information are available in the Supplementary Material in the “mtx” and “pfinder” folders respectively.

### Tree inferences

We carried out a maximum likelihood tree search using RAxML-HPC v.8 (Stamatakis, 2014). We incorporated a backbone tree with some node constraints, especially at the family level, based on other evolutionary hypotheses available in the literature (e.g., (Armbruster et al., 2015; Tagliacollo et al., 2016; Lujan, et al., 2018 Melo et al., 2022; Reis and Lehmann, 2022). The backbone tree and its respective node constraints are available in the Supplementary Material in the “mtx” folder. These constraints were necessary due to the low phylogenetic signal for resolving deep node relationships. Unfortunately, alternative genomic markers like UCEs (Faircloth et al., 2012) that are more suitable for inferring deeper node relationships were not available for enough NFF taxa to be included in the matrix. We designated the two lamprey (Agnatha) species *Geotria australis* and *Mordacia lapicida* as outgroup taxa and rooted the tree at their most recent common ancestral node. RAxML estimated all free model parameters and optimized the likelihood of the final tree under the GAMMA model using its rapid hill-climbing mode. To assess tree support, we conducted 100 bootstrap replicates on the resulting likelihood tree topology. RAxML analyses were performed on the CIPRES Gateway portal (Miller et al., 2010). Since some analyses are compatible with fully bifurcating trees exclusively and will not be possible given trees with polytomies, we include both fully resolved trees and trees with poorly supported nodes (bootstrap values below 70%) collapsed in our Supplementary Material in the “phy” folder. We advise researchers pursuing downstream work to use the high-confidence trees where possible, and only use the fully resolved trees if their given approach is incompatible with polytomies.

### Divergence time estimates

Estimating divergence times concurrently with topology using Bayesian inference would be challenging due to the large size of the matrix and the number of terminals. Additionally, given the geological conditions of the Neotropics, fish fossils are typically quite rare, leaving us with few reliable primary calibrations. As an alternative, we used the congruification method implemented in the R package geiger v2 (Pennell et al., 2014) to estimate divergence times using a reference time-calibrated tree as a source of secondary calibrations (Eastman et al., 2013; Pennell et al., 2014). In brief, divergence times of a target tree (with branch lengths in units of nucleotide substitutions) can be estimated by taking any divergence times also found in the reference tree as secondary calibrations. For this, we selected a published global time-calibrated phylogeny of ray-finned fishes based on 1,105 selected phylogenomic markers from 303 extant species representing 66 or 72 recognized ray-finned fish orders, and including 31 fossil calibrations (Hughes et al., 2018). As an additional source of secondary calibrations among cartilaginous fish, we used the shark tree of life based on 10 fossil calibrations (Stein et al., 2018). Divergences shared between our tree and these two reference trees were identified using the congruify.phylo () function in *geiger* (Pennell et al., 2014) in R (R Core TeamR, 2023). These divergence times were then used to calibrate nodes in our tree using treePL (Smith and O'Meara, 2012). We first initialized the treePL optimization by setting thorough and prime to TRUE, and then used the default priming step to optimize parameters when estimating divergence times. The suggested optimized parameters included opt = 1, optad = 1, and optcvad = 4. The treePL input files with all calibration points and parameter settings can be found in our Supplementary Materials in the “phy” folder.

## Results

### Species relationships inferred from RAxML

**FIGURE 1:**
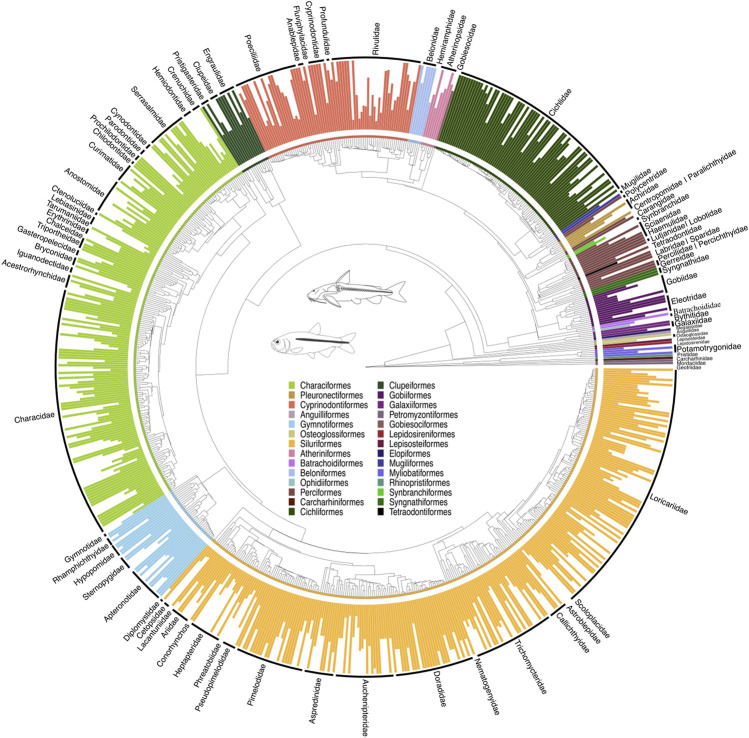
A time-calibrated phylogeny of 3,167 NFF species (or 51% of NFF) allocated to 741 genera (or 86% of NFF), illustrating evolutionary relationships among genera across major lineages. This tree was estimated using RAxML-HPC and time-calibrated through congruification (Eastman et al., 2013) employing divergence tree estimates from Hughes et al. (2018). The tree resulted from alignments of 51 orthologous markers, totaling 43,850 bp, with 21,223 sequences and 5,984 terminals. Vertical bars indicate the complete species per genus represented on the tree. All GenBank accession numbers, alignments, species-level relationships, divergence clade estimates and species representativeness percentages are provided in the Supplementary Material. This phylogeny, as well as one with nodes bearing bootstrap values below 70% collapsed, is available as a Newick string in the Supplementary Material and can be found in the “phy” folder.

**FIGURE 2:**
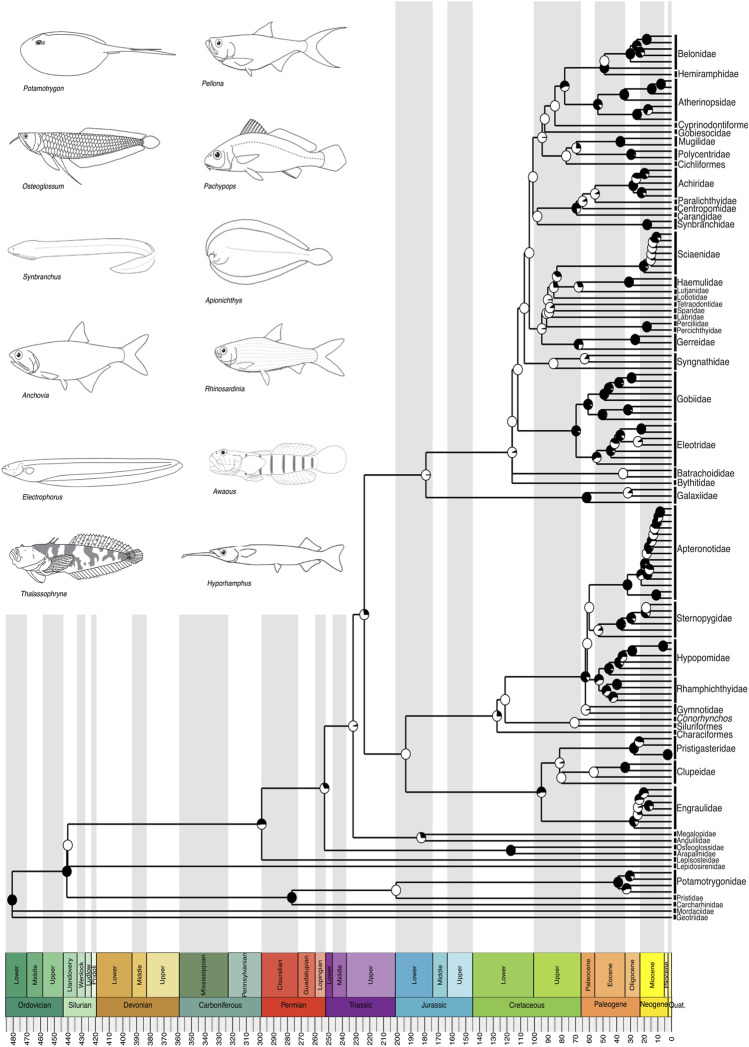
A time-calibrated phylogeny of Neotropical Freshwater Fishes (NFF) illustrating evolutionary relationships among genera from all recognized families, except within Characiformes (shown in detail in Figure 3), Siluriformes (shown in detail in Figure 4), Cichliformes (shown in detail in Figure 5), and Cyprinodontiformes (shown in detail in Figure 6). Divergence time estimates indicate that the evolutionary history of the NFF has its roots in the Ordovician, around 480 million years ago (mya), a period of high diversification in the oceans, particularly among agnathans. Pie charts on nodes indicate bootstrap support, with the proportion of black space corresponding to the percentage of bootstrap replicates in support. Full black circles represent 100% support. All GenBank accession numbers, alignments, species-level relationships, divergence clade estimates and species representativeness percentages are provided in the Supplementary Material. This phylogeny, as well as one with nodes bearing bootstrap values below 70% collapsed, is available as a Newick string in the Supplementary Material and can be found in the “phy” folder.

Here we present the most species-rich NFF tree to date, comprising 5,984 terminals and 3,167 species across 741 genera (Figure 1). The majority of the phylogenetic relationships we recover are consistent with published molecular studies (Oliveira et al., 2011; Near et al., 2012; Sullivan et al., 2013; Tagliacollo et al., 2016; Betancur-R et al., 2017; Hughes et al., 2018; Ilves et al., 2018; Betancur-R et al., 2019), as expected given that much of the data underlying our novel supermatrix was submitted to GenBank as part of numerous past phylogenetic efforts. Relationships among the four largest clades of the NFF, Characiformes, Siluriformes, Cichliformes, and Cyprinodontiformes corroborate the current consensus in fish classification (Dornburg and Near, 2021). Our tree estimates positions Lepisosteiformes (i.e., gars) as the sister group to Teleostei, with the clade Euteleostei as the sister group to Elopiformes (Anguillidae, Megalopidae), Osteoglossiformes (Arapaimidae, Osteoglossidae), and Otocephala (Figure 2). Within Otocephala, our findings support a close relationship between the Clupeiformes and Characiphysae, with Characiformes forming the sister group to Siluriformes and Gymnotiformes (Figure 2).

**FIGURE 3:**
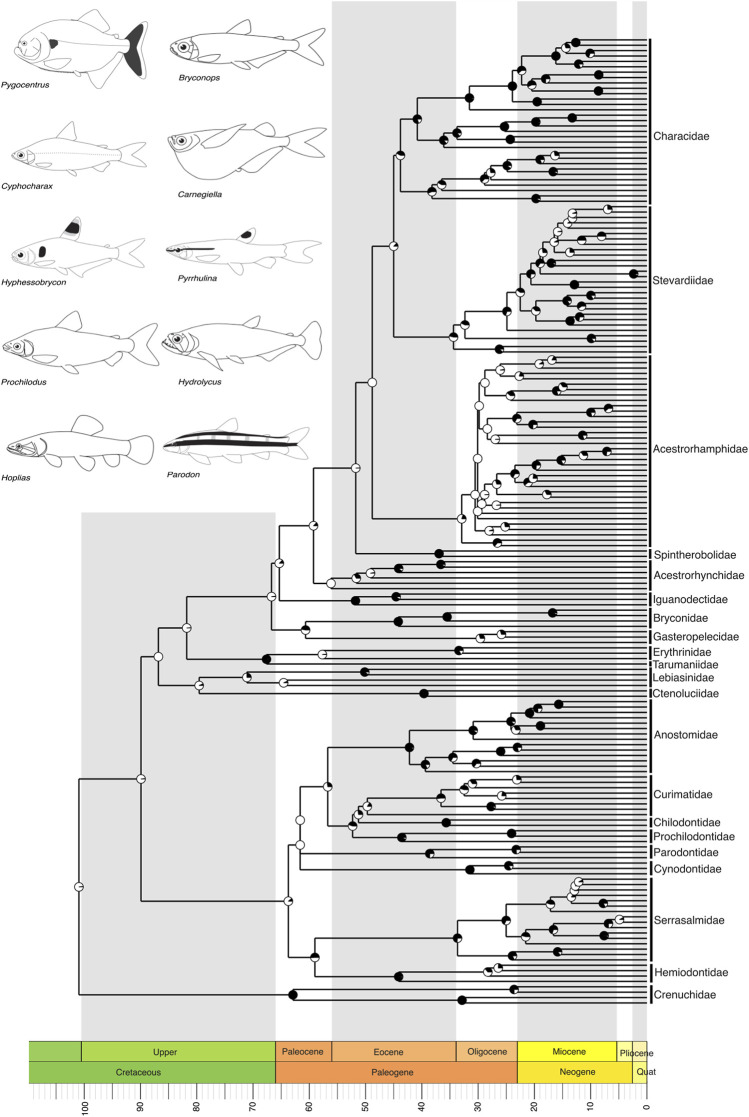
A time-calibrated phylogeny of Characiformes, the second-largest clade in the Neotropics, illustrating evolutionary relationships among genera of all currently recognized families. Divergence times place the most recent common ancestor of Neotropical characiforms at the boundary of the Upper Cretaceous, around 100 million years ago (mya). Pie charts on nodes indicate bootstrap support, with the proportion of black space corresponding to the percentage of bootstrap replicates in support. Full black circles represent 100% support. All GenBank accession numbers, alignments, species-level relationships, divergence clade estimates and species representativeness percentages are provided in the Supplementary Material. This phylogeny, as well as one with nodes bearing bootstrap values below 70% collapsed, is available as a Newick string in the Supplementary Material and can be found in the “phy” folder.

Within the first major NFF clade, Characiformes, which includes the piranha as well as over a thousand small tetras and darters, our tree includes only representatives of the suborder Characoidei, recapitulating the monophyly of families and several well-established relationships at genus level (Oliveira et al., 2011; Tagliacollo et al., 2012; Abe et al., 2014; Thomaz et al., 2015; Melo et al., 2022). These relationships include Crenuchidae as the sister group to all other characiforms, the monophyly of Anostomoidea (*sensu* Vari, 1983; Vari, 1983; i.e., Curimatidae, Prochilodontidae, Anostomidae, and Chilodontidae, but see Betancur et al., 2019), a sister-group relationship between Ctenoluciidae and Lebiasinidae, and the monophyly of Characidae (*sensu* Oliveira et al., 2011, but see Melo et al., 2024), including the genus *Amazonspinther* as the sister group to all remaining characids (Figure 3).

**FIGURE 4:**
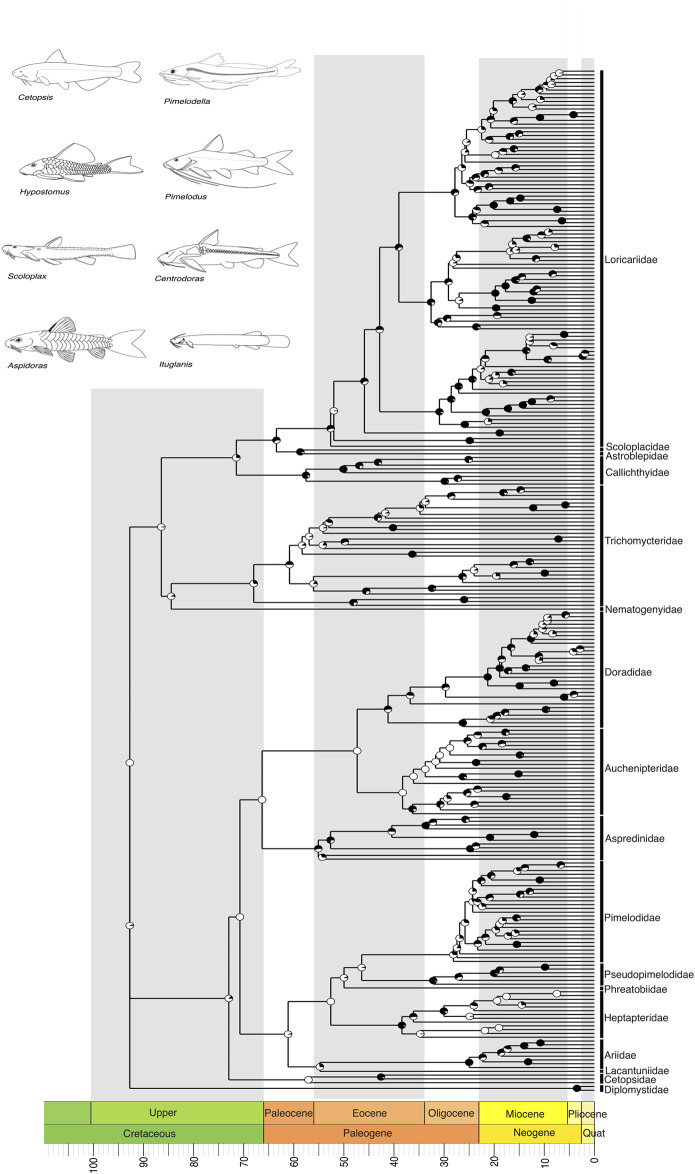
A time-calibrated phylogeny of siluriformes, the largest clade of Neotropical Freshwater Fishes (NFF), illustrating evolutionary relationships among genera of all currently recognized Neotropical families. Divergence time estimates suggest that the most recent common ancestor of Neotropical siluriformes and some of its families (e.g., stem clades: Diplomystidae, Cetopsidae, Nematogenyidae, Trichomycteridae) arose in the Upper Cretaceous, around 100 to 66 million years ago (mya). Pie charts on nodes indicate bootstrap support, with the proportion of black space corresponding to the percentage of bootstrap replicates in support. Full black circles represent 100% support. All GenBank accession numbers, alignments, species-level relationships, divergence clade estimates and species representativeness percentages are provided in the Supplementary Material. This phylogeny, as well as one with nodes bearing bootstrap values below 70% collapsed, is available as a Newick string in the Supplementary Material and can be found in the “phy” folder.

Within the second major clade, Siluriformes, comprising over a thousand catfishes ranging from miniatures such as *Scoloplax* to the six-foot riverine predator *Brachyplatystoma*, our tree indicates that Diplomystidae forms the sister group to all other catfishes, which are further divided into two primary clades (*sensu* Sullivan et al., 2006) Loricarioidei and Siluroidei (Figure 4). Within Siluroidei, several well-established relationships were corroborated, such as the close relationships between Doradidae, Aspredinidae, and Auchenipteridae, as well as the grouping of Pseudopimelodidae, Pimelodidae, Phreatobiidae, Heptapteridae and *Conorhynchos* (Figure 4). Within Loricarioidei, our tree indicates that Nematogenyidae is the sister group to the remaining families (Figure 4), and Trichomycteridae is sister to remaining loricarioids, with Callichthyidae being sister to advanced loricarioids and Loricariidae, and Astroblepidae forming a sister group to Scoloplacidae (Figure 4). A point to note is that within Trichomycteridae, the subfamily Vandelliinae is not closely related to Tridentinae and Stegophilinae. This result must be interpreted with caution. Several independent studies have shown a close relationship between these three clades including through genomic data (de Pinna, 1998; Ochoa et al., 2017; Ochoa et al., 2020), which are important to our understanding of the origins of parasitism in the Trichomycteridae clade.

**FIGURE 5:**
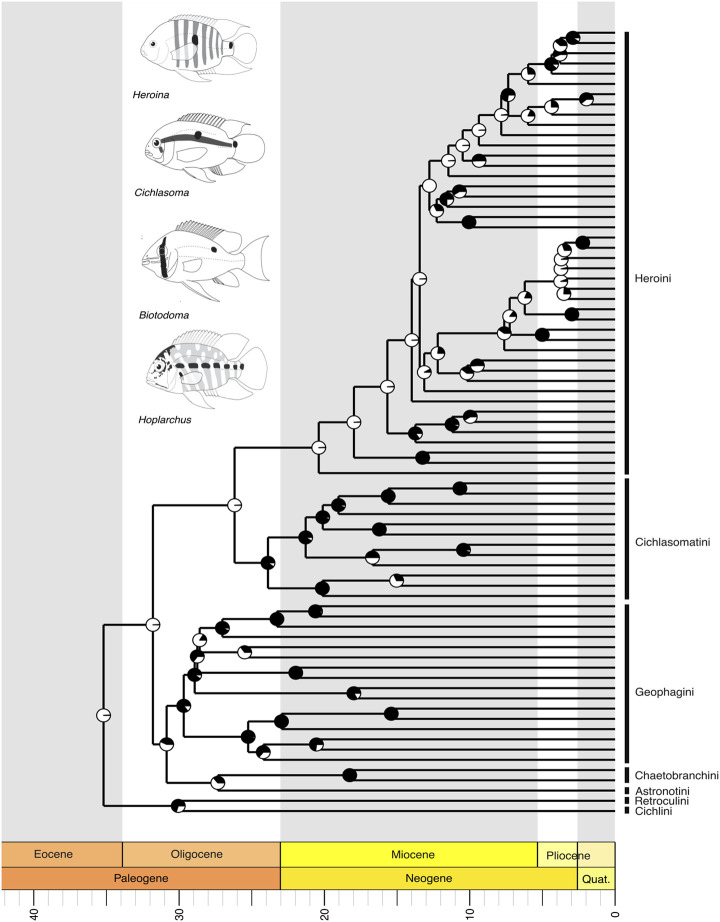
A time-calibrated phylogeny of the Neotropical Cichlidae, showing evolutionary relationships among genera of all currently recognized Neotropical families. Divergence time estimates indicate that Cichlidae is the youngest of the four largest clades of Neotropical Freshwater Fishes (NFF), with the most recent common ancestor evolving in the Cenozoic, during the Eocene, around 40 million years ago (mya). Pie charts on nodes indicate bootstrap support, with the proportion of black space corresponding to the percentage of bootstrap replicates in support. Full black circles represent 100% support. All GenBank accession numbers, alignments, species-level relationships, divergence clade estimates and species representativeness percentages are provided in the Supplementary Material. This phylogeny, as well as one with nodes bearing bootstrap values below 70% collapsed, is available as a Newick string in the Supplementary Material and can be found in the “phy” folder.

Within the third major clade, Cichliformes, including cichlid fishes like the peacock bass and the freshwater angelfish and discus, our tree supports the monophyly of the seven proposed tribes (*sensu* López-Fernández et al., 2010; Ilves et al., 2018). The relationships we recover suggest that Cichlini is the sister group to Retroculini, and together they represent the earliest-diverging lineages among Neotropical cichlids (Figure 5). Astronotini and Chaetobranchini were recovered as sister groups, collectively forming a sister clade to Geophagini (Figure 5). This latter clade is related to a large group consisting of Cichlasomatini, which is sister to Heroini, a lineage that includes representatives in Central America (Figure 5). Within Heroini, the biogeographical distribution patterns of species suggest that the tribe originated in South America, with subsequent colonization of Central America and the Caribbean occurring through more than one independent event during the Neogene (Figure 5).

**FIGURE 6:**
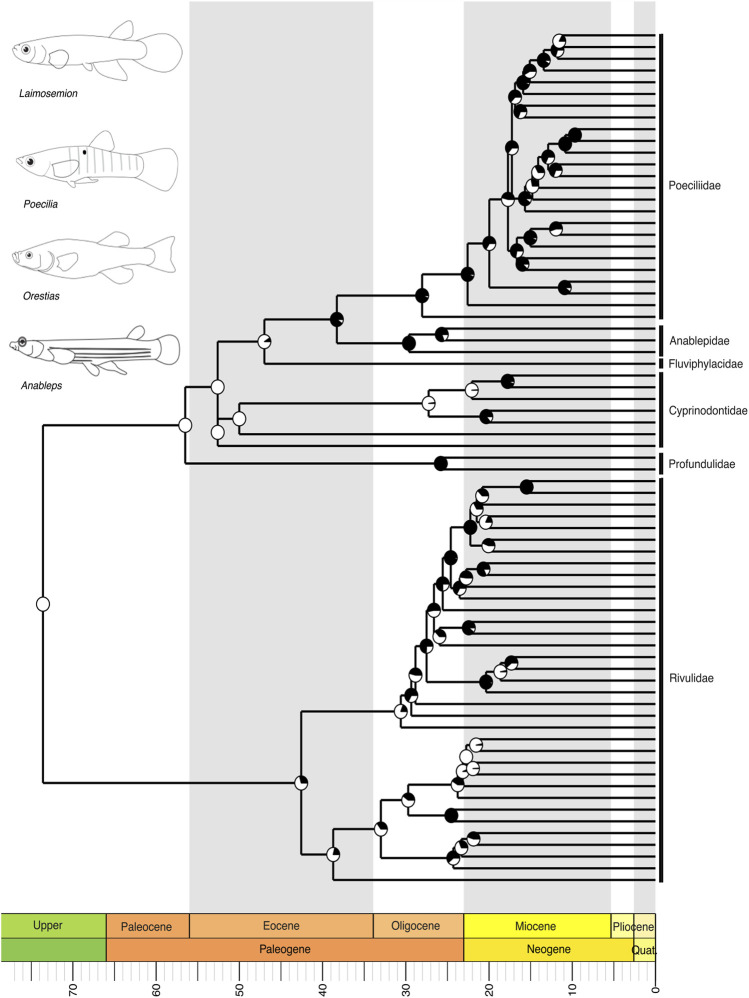
A time-calibrated phylogeny of Cyprinodontiformes illustrates the evolutionary relationships among Neotropical clades including Rivulidae, Profundulidae, Cyprinodontidae, Poeciliidae, Anablepidae, and Fluviphylacidae, as well as their respective genera. Divergence estimates place the origin of this clade in the Upper Cretaceous, with the most recent common ancestor of Rivulidae, Cyprinodontidae, and Fluviphylacidae appearing in the Eocene, and that of Profundulidae, Poeciliidae, and Anablepidae in the Oligocene. Pie charts on nodes indicate bootstrap support, with the proportion of black space corresponding to the percentage of bootstrap replicates in support. Full black circles represent 100% support. All GenBank accession numbers, alignments, species-level relationships, divergence clade estimates and species representativeness percentages are provided in the Supplementary Material. This phylogeny, as well as one with nodes bearing bootstrap values below 70% collapsed, is available as a Newick string in the Supplementary Material and can be found in the “phy” folder.

Within the fourth major NFF clade, Cyprinodontiformes, including the livebearing guppies and killifish, our tree revealed a close relationship between two major clades: one comprising the family Rivulidae and another consisting of Profundulidae, Cyprinodontidae, Fluviphylacidae, Anablepidae, and Poeciliidae (Figure 6). In this latter clade, we recovered Profundulidae as the sister group to all other families, followed by Cyprinodontidae as sister to the remaining clades (Figure 6). Poeciliidae, the largest group within this clade, was recovered as the sister group to Anablepidae, and together they formed a sister group relationship with Fluviphylacidae (Figure 6). As seen in Cichliformes, the biogeographical distribution patterns suggest that this group originated in South America, with subsequent colonization of Central America.

### Time-calibrated tree

Our divergence time estimates align closely with the established timeline of NFF evolution found in our reference phylogenies, Hughes et al. (2018) and Stein et al. (2018), and other published independently-estimated divergence times accessible through TimeTree.org (McMahan et al., 2013; Kappas et al., 2016; Fontenelle et al., 2021; Melo et al., 2022). The NFF earliest lineages to diversify, the jawless lamprey (Agnatha) species, diverged from other clades in the Paleozoic, specifically during the Ordovician, approximately 480 million years ago (mya) (Figure 2). This divergence aligns with the Great Ordovician Biodiversification (GOBI) event, during which vertebrates, particularly fishes, emerged and reached their peak diversification in the Devonian (Servais et al., 2010). The three gnathostome vertebrate lineages with extant freshwater species in the Neotropics—Chondrichthyes, Sarcopterygii, and Actinopterygii—share a most recent common ancestor from the Silurian, with a subsequent split in the Permian, between 299 and 252 mya (Figure 2). The Permian period was marked by the formation of Pangaea and one of the largest mass extinction events in Earth’s history (Erwin et al., 2002), creating new evolutionary opportunities for surviving lineages to diversify, including the Actinopterygian ray-finned fishes, which today dominate freshwater environments worldwide.

The Actinopterygii, encompassing all four major clades of the NFF (Characiformes, Siluriformes, Cichliformes, and Cyprinodontiformes) as well as the electric eel and other electric fishes (Gymnotiformes), diverged from their sister group the Sarcopterygian lobe-finned fishes during the Silurian, with most families within Actinopterygii diversifying during the Paleogene, specifically between 66 and 23 mya (Figure 2). This period was characterized by significant geomorphological changes in South America, including the uplift of the Andes, which rearranged river basins, particularly in the Western Amazon (Hoorn et al., 2010; Wilkinson et al., 2010; Albert et al., 2018) and triggered marine incursions along the sub-Andean Foreland (Bloom et al., 2011). At 23 mya, the Miocene epoch saw the formation of the Pebas system, a network of long-lived lakes and wetlands in western Amazonia (Hoorn et al., 2010; Boschman et al., 2023). Most modern lineage diversification within the NFF occurred during this interval, suggesting that the Pebas system, along with the shift in the Amazon River from its original Northward drainage to its current mouth at Belém in Eastern Brazil, played an important role in shaping one of the richest freshwater vertebrate faunas on Earth (Albert et al., 2018; Albert et al., 2021).

We recover the crown of the Characiformes in the Upper Cretaceous, with most families evolving during the Paleogene, particularly in the Eocene (55–33 mya) (Figure 3). The Siluriformes also originated in the Upper Cretaceous, with the majority of families diversifying during the Paleogene, primarily in the Eocene (Figure 4). Fossil records indicate that both Characiformes and Siluriformes were already established in the continental waters of South America during the Upper Cretaceous (Alves et al., 2019). This is further supported by the modern distribution of Characiformes in Africa and South America, and Siluriformes globally, except in Antarctica (although an Eocene–Oligocene fossil has been reported from Antarctica; (Grande and Eastman, 1986), suggesting that dispersal likely occurred during the era of the Pangaea supercontinent.

The Neotropical cichlids of the subfamily Cichlinae (Cichlidae), the youngest of the major NFF clades, evolved approximately 40 million years ago during the Cenozoic (Figure 5). Within this clade, all South American genera originated in the Miocene, while a few Central American genera emerged later during the Pliocene-Pleistocene (Figure 5). The origins of Central American cichlids remain a point of debate (Holden and Dietz, 1972; MacPhee, 1999; Bacon et al., 2015), likely involving multiple colonization and diversification events associated with the Caribbean plate margins (Tagliacollo et al., 2017). The Cyprinodontiformes of the Neotropics also diverged in the Upper Cretaceous, with the most recent common ancestors of Rivulidae, Cyprinodontidae, and Fluviphylacidae diversifying in the Eocene, while those of Profundulidae, Poeciliidae, and Anablepidae diverged in the Oligocene (Figure 6). All phylogeny files are available in easily viewable and editable Newick format in the Supplementary Material in the “phy” folder. Divergence estimates for NFF families are provided in Supplementary Table S1.

## Discussion

Constructing a comprehensive phylogenetic tree of NFF species from public data repositories such as GenBank is important in both scientific and conservation contexts (Winter et al., 2013; Tucker et al., 2017). Scientifically, the tree presented here provides a holistic view of the NFF biodiversity, unraveling evolutionary relationships and establishing a comparative framework to test macroevolutionary hypotheses in the Neotropics. It will also form a baseline for continuous updates as new sequences become available through the collective efforts of taxonomists and evolutionary biologists, advancing our understanding of one of the Earth’s most diverse biotas (e.g., Antonelli et al., 2017). Maintaining an accurate timed phylogeny also benefits databases such as TimeTree.org, allowing researchers who are not phylogenetic experts to export phylogenies or summaries of divergence times for their own downstream analyses (Kumar et al., 2022).

This phylogeny also plays an important role in conservation efforts. It will inform targeted initiatives based on evolutionary distinctiveness and phylogenetic shape and rate metrics, and will contribute to threat assessments among the NFF species (Weber et al., 2017; Albert et al., 2020). Given that continental freshwaters harbor diverse yet fragile ecosystems threatened by human activities, prioritizing geographic areas with the highest number of coexisting evolutionary lineages, including those with threatened species, can significantly enhance conservation efforts for NFF taxa (Tagliacollo et al., 2021; Dagosta et al., 2021).

Maintaining this comprehensive NFF tree presents challenges due to the ongoing discovery of new species, taxonomic revisions, and advancements in genomic sequencing technologies (Remec et al., 2021). Consequently, community-driven efforts are crucial for updating the tree, encompassing the inclusion of newly-described taxa, review of taxonomic changes, and the integration of new DNA markers generated by next-generation sequencing technologies such as UCEs (Faircloth et al., 2012) and exons (Bragg et al., 2016). Over the years, new DNA sequences will be generated as methodologies improve, allowing for the continuous refinement of evolutionary hypotheses. Future studies will also incorporate phylogenomic data, improving node support and removing some tree constraints that were necessary due to the lack of sufficient phylogenetic signal to recover deeper node evolutionary relationships.

Using publicly available sequences for research comes with inherent limitations, including misidentifications of taxa, where sequences may be attributed to the wrong species due to errors in specimen identification, and incomplete coverage in the alignments, which can affect the accuracy and comprehensiveness of analyses. While misidentified sequences are common on public sequence repositories like GenBank (Li et al., 2018), we mitigated their impact through rigorous manual evaluation by multiple experts in Neotropical ichthyology, and we will continue to review public data by the community and evaluate the reliability of sequences during future updates to the tree. As these updates unfold, the tree will expand in the number of both taxa and molecular markers, requiring the utilization of bioinformatic tools to standardize and streamline such updating processes. Because public databases can often contain sequences that are incomplete or represent only certain regions of the genome, which can impact the accuracy and comprehensiveness of analyses (Li et al., 2018), researchers should always be aware of these limitations and biases when using publicly available DNA sequences and exercise caution in interpreting and generalizing results derived from such data. Incorporating multiple data sources and rigorous validation processes can help mitigate these issues to some extent.

One possible alternative is to continue developing methodologies of phylogenetic placement which allow us to determine the phylogenetic position of new species without remaking the entire tree (e.g., Matsen et al., 2010). Importantly, phylogenetic placement techniques require less time and computational power than full Bayeseian or likelihood-based tree inferences and can be implemented collaboratively on platforms connected to computer servers across different locations. While this strategy provides a more efficient means of performing small, localized updates to the phylogenetic tree, it will still periodically be necessary to rebuild the entire tree to incorporate all new taxa and genomic markers, which will be a computationally intensive process. This only underscores the importance of investing in high powered computational infrastructure as the field evolves.

The ichthyological research community is invited to embark on a collaborative journey to enhance the phylogenetic tree of NFF species. With a dedication to continuous data contribution, available online in repositories such as GenBank, periodic taxonomic reviews, and methodological advancements, the community can collectively refine and expand this initial effort to elucidate the evolutionary relationships among NFF taxa. Emphasizing data sharing, interdisciplinary collaboration, and community engagement will foster a clear understanding and application of these phylogenetic insights in future studies. The curation of public repositories, development of new methods, and interdisciplinary collaboration ensure a robust and accessible framework for ongoing research, aiming to reveal the intricate processes and patterns of formation of the diverse NFF taxa. A proactive approach to address data gaps solidifies the commitment to advancing our knowledge of the evolutionary relationships of NFF species, supporting empirical means for biodiversity conservation plans, and contributing to global scientific endeavors uncovering the origins and diversification of freshwater vertebrate faunas in the Neotropics.

## Conclusion

This study presents the first comprehensive, time-calibrated phylogeny of NFF species, based on a supermatrix of 51 genes with a total of 43,850 bp for 5,984 specimens in 3,167 species representing 50% of all valid NFF species and 741 or 86% of all NFF genera. This supermatrix reflects three decades of molecular research by the ichthyological community by drawing on sequence data deposited in GenBank in hundreds of separate studies. While standing out as the most species-rich dataset of NFF taxa assembled to date, the accuracy of this phylogeny is affected by the representation of genes and taxa included in previous investigations, with known biases in the representations of fish species by clade (e.g., family), traits (e.g., adult body size), geographic and ecological ranges (e.g., investigator access), and conservation status. Despite these shortcomings, this study establishes an initial framework for fauna-wide investigations of macroevolutionary and macroecological patterns, acknowledging the need for continual updates as science progresses. Our commitment as a scientific community to regular updates ensures its increasing accuracy and relevance, aligning with advancing ichthyological knowledge. This study underscores the importance of collaborative efforts and unlocks avenues for future research in historical biogeography, macroevolution, and macroecology. By fostering active collaboration and embracing scientific inquiry, we hope this assembled tree serves as a start point for more efforts to protect freshwater fishes, marking a stride toward sustainable biodiversity conservation.

## Data Availability

The original contributions presented in the study are included in the article/Supplementary Material, further inquiries can be directed to the corresponding author.
